# A Review of Myoelectric Control for Prosthetic Hand Manipulation

**DOI:** 10.3390/biomimetics8030328

**Published:** 2023-07-24

**Authors:** Ziming Chen, Huasong Min, Dong Wang, Ziwei Xia, Fuchun Sun, Bin Fang

**Affiliations:** 1 Laboratory for Embedded System and Intelligent Robot, Wuhan University of Science and Technology, Wuhan 430081, China; ziming_chen@wust.edu.cn (Z.C.); mhuasong@wust.edu.cn (H.M.); 2 Institute for Artificial Intelligence, State Key Lab of Intelligent Technology and Systems, Department of Computer Science and Technology, Beijing National Research Center for Information Science and Technology, Tsinghua University, Beijing 100084, China; 3 School of Engineering and Technology, China University of Geosciences, Beijing 100083, China

**Keywords:** myoelectric control, intention recognition, control strategy, functionality-augmented prosthetic hands, user burden reduction

## Abstract

Myoelectric control for prosthetic hands is an important topic in the field of rehabilitation. Intuitive and intelligent myoelectric control can help amputees to regain upper limb function. However, current research efforts are primarily focused on developing rich myoelectric classifiers and biomimetic control methods, limiting prosthetic hand manipulation to simple grasping and releasing tasks, while rarely exploring complex daily tasks. In this article, we conduct a systematic review of recent achievements in two areas, namely, intention recognition research and control strategy research. Specifically, we focus on advanced methods for motion intention types, discrete motion classification, continuous motion estimation, unidirectional control, feedback control, and shared control. In addition, based on the above review, we analyze the challenges and opportunities for research directions of functionality-augmented prosthetic hands and user burden reduction, which can help overcome the limitations of current myoelectric control research and provide development prospects for future research.

## 1. Introduction

Motor neurons integrate inputs from the central nervous system (CNS) and incoming feedback and transform them into neural drive to the muscles [1]. In simple terms, motor neurons release action potentials to the muscles, translating these neural commands into force and movement. A motor unit (MU) is a functional unit that consists of a motor neuron and the muscle fibers innervated by it. It is the smallest neurologically controlled unit that describes the process of muscle contraction. The sum of action potentials in the muscle fibers of an MU represents the motor unit action potential (MUAP) [2].

The electromyographic (EMG) signal, which can be captured by sensing devices, is obtained through the convolution of each motor neuron’s pulse sequence with MUAP [3]. In reality, the EMG signal is a composite signal resulting from the superposition of multiple MUAP sequences [4]. Therefore, the EMG signal directly reflects the discharge characteristics of the MUs within the measured muscle. The amplitude of EMG signals typically ranges between ±5000 μV, while the frequency range is usually between 6 and 500 Hz, with the main frequency power falling within the range of 20 to 150 Hz [5].

Based on the placement of sensing devices, the measured EMG signals can be divided into intramuscular electromyography (iEMG) and surface electromyography (sEMG). iEMG is obtained by implantable sensing devices placed inside the subject’s body. Its advantage lies in its ability to capture EMG signals from specific muscle locations, which is more conducive to exploring the relationship between muscle activation and task execution. However, a drawback is that implantable sensing devices inevitably cause physical harm to the subject’s limb and raise concerns about long-term adaptability. On the other hand, sEMG is acquired through sensing devices placed on the surface of the skin. Although it can only capture combined EMG signals from muscle groups, it has been favored in research on myoelectric prosthetic hand control due to its ease of acquisition and rich information content.

The concept of myoelectric control was first proposed in 1948, but it was not until the 1960s that myoelectric prosthetics had a significant clinical impact [6]. Despite decades of satisfying laboratory research results, the high abandonment rate of prosthetic hands has not been improved, which is mainly due to amputees not being able to receive intuitive and natural myoelectric control. The development stages of myoelectric control can be summarized as (1) switch control strategy; (2) proportional control strategy; (3) pattern recognition control strategy; and (4) simultaneous and proportional control strategy:

- The switch control strategy is a simple technique that uses smoothed and rectified sEMG signals and predefined thresholds to achieve single-degree-of-freedom control of prosthetic hands, such as grasp or wrist rotation. Specifically, the principle of this strategy is to establish a mapping between sEMG amplitude and activation of prosthetic hand movement. If the amplitude is greater than a manually preset threshold, the prosthetic hand will execute the action at a constant speed/force;
- The proportional control method can achieve variable speed/force movements of the prosthetic hand based on the proportion of user input signals. The proportional control strategy establishes a mapping between sEMG amplitude and the degree of movement of the prosthetic hand, where the description variable of the degree of movement can be force, speed, position, or another mechanical output;
- Pattern recognition technology is a method based on feature engineering and classification techniques and is currently a research hotspot in myoelectric control. The principle of pattern recognition control strategy is that similar sEMG signal features will be reproduced in experiments with the same action pattern. These features can be used as the basis for distinguishing different action patterns, thereby recognizing a wider variety of action patterns than the input channel number. The pattern recognition control strategy simplifies the representation of hand movements, effectively reduces the difficulty of the task, and significantly improves the accuracy of traditional motion intent recognition;
- The simultaneous and proportional control strategy aims to capture the entire process of the user’s execution of hand movements, including the different completion stages of a single action and the transition stages between different actions, which is a more complex and dynamic process. Compared to the above control strategy, the multi-degree-of-freedom simultaneous proportional control strategy does not rely on pre-set action patterns but instead estimates the hand state at a single moment in real-time based on regression methods, such as joint angles, positions, or torques. This feature allows users to control the myoelectric prosthetic hand more intuitively and naturally, making it a new research hotspot in the field of myoelectric control in recent years.

Despite the progress made in the past decade in areas such as biocompatible electrodes, surgical paradigms, and mechatronics integration [7], the development of current myoelectric prosthetic hand control schemes have been hindered by limitations in the field of motion intention recognition, resulting in a lack of intuitive and robust human–machine interfaces. Previous works have reviewed various aspects of research progress, including methods for predicting continuous upper limb movements based on sEMG [8], the application of deep learning in multi-task human–machine interaction (HMI) based on sEMG [9], and various performance indices in myoelectric control [10]. Existing myoelectric control research for prosthetic hand interaction mainly focuses on intention recognition and control strategy, aiming to accurately decode human intention through recognition algorithms and drive the prosthetic hand to execute the intent through control algorithms. However, further research is supposed to develop myoelectric control schemes for complex daily manipulation scenarios [11]. There is still a lack of attention to the challenges and opportunities that may be encountered in the development of myoelectric prosthetic hand manipulation capabilities.

The main contributions of this article are as follows: (1) We provide a comprehensive review of the basic concepts in myoelectric control, with a focus on mapping human intention to control parameters; (2) We divide the current state of myoelectric control research into two aspects for a thorough review, namely, intention recognition and control strategy; (3) We discuss the current challenges and future research directions of prosthetic hand manipulation, aiming to provide a novel perspective for the advancement of the myoelectric control field.

The remainder of the article is organized as follows: Section 2 presents the basic concepts of myoelectric control; Section 3 presents current research status, including advances in intention recognition and control strategy research; Section 4 presents challenges and opportunities in the future of myoelectric control; Section 5 presents the conclusions of the article.

## 2. Basic Concepts of Myoelectric Control

**Figure 1:**
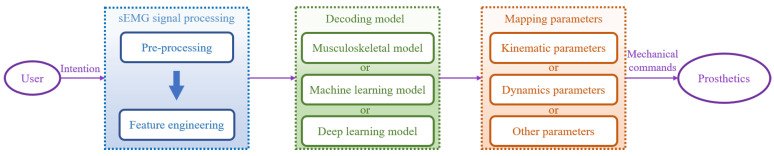
Basic concepts of myoelectric control.

In this section, we introduce the basic concepts of myoelectric control, which include sEMG signal processing, decoding models, and mapping parameters. The approach to myoelectric control involves first characterizing the user’s motion intention as specific physical parameters, which are then translated into control commands to achieve motion control of the prosthetic hand. The specific steps are illustrated in Figure 1. A summary of the related research for each section is provided in Table 1.

### 2.1. sEMG Signal Processing

sEMG signal analysis refers to a series of processing steps applied to the acquired human sEMG signals using signal acquisition devices. The primary objective is to eliminate irrelevant noise unrelated to the intended movements while retaining as many useful features as possible. This process aims to accurately identify the user’s intended movements.

#### 2.1.1. Pre-Processing

Common mode interference signals can have an impact on the sEMG signals of the human torso [53], such as 50 Hz power line interference, line voltage, and contamination from myocardial electrical activity [54]. Additionally, inherent instability exists within the sEMG signals themselves. The sEMG signals contain noise within the 0–20 Hz frequency range, influenced by the firing rate of MUs, while high-frequency signals exhibit a lower power spectral density [55]. Therefore, to improve the quality of sEMG signals, commonly used filters are employed to remove frequency components below 20 Hz and above 500 Hz, as well as interference signals at around 50 Hz.

Applying only filtering processing to the raw signals and inputting them into the decoding model can maximize the retention of useful information from the human signals. This approach aims to enhance the performance and practicality of the intent recognition system for real-world applications in myoelectric prosthetic hand systems. However, achieving significant accuracy in intent recognition solely through filtering the sEMG signals requires reliance on a decoding model with feature learning capabilities. The hierarchical structure of the decoding model transforms simple feature representations into more abstract and effective ones [56].

#### 2.1.2. Feature Engineering

Traditional feature engineering for analyzing EMG signals can typically be categorized into three types: time-domain, frequency-domain, and time–frequency-domain features [57]. Time-domain features extract information about the signal from its amplitude, frequency-domain features provide information about the power spectral density of the signal, and time–frequency-domain features represent different frequency information at different time positions [58]. Frequency-domain features have unique advantages in muscle fatigue recognition. However, a comparative experiment based on 37 different features showed that frequency-domain features are not well suited for EMG signal classification [59]. Time–frequency-domain features are also limited in their application due to their inherent computational complexity. Time-domain features are currently the most popular feature type in the field of intent recognition. Therefore, we mainly focus on single and combined features based on time-domain features.

The feature engineering techniques allow for the mapping of high-dimensional sEMG signals into a lower-dimensional space. This significantly reduces the complexity of signal processing, retaining the useful and distinguishable portions of the signal while eliminating unnecessary information. However, due to the stochastic nature of sEMG signals and the interference between muscles during movement, traditional feature engineering inevitably masks or overlooks some useful information within the signals, thereby limiting the accuracy of intent recognition. Additionally, since sEMG signals contain rich temporal and spectral information, relying on specific and limited feature combinations may not yield the optimal solution [45]. Moreover, a universally effective feature has not been identified thus far, necessitating further exploration of features that can make significant contributions to improving intent recognition performance.

### 2.2. Decoding Model

**Figure 2:**
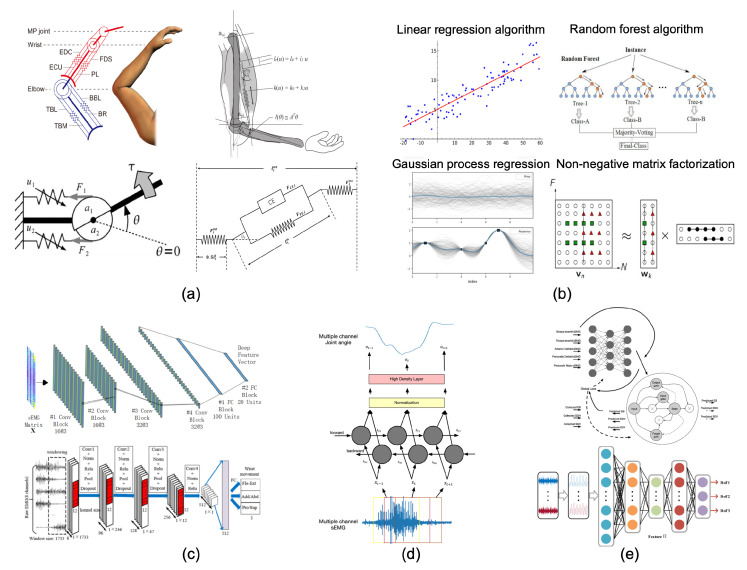
Application examples of decoding models: (**a**) Example of musculoskeletal model applications [23,60,61,62]. (**b**) Examples of traditional machine learning model applications. (**c**) Example of CNN-based model applications. [46,49]. (**d**) Example of RNN-based model applications [37]. (**e**) Example of hybrid-structured model applications [40,41].

Decoding models serve as a bridge between user motion intentions and myoelectric prosthetic hand control commands, playing a significant role in motion intention recognition schemes. The purpose of a decoding model is to represent the linear or nonlinear relationship between the inputs and outputs of the motion intention recognition scheme, which can be achieved through either establishing an analytical relationship or constructing a mapping function. The former are known as model-based methods, while the latter are referred to as model-free methods [8], which include traditional machine learning algorithms and deep learning algorithms. In this section, we discuss the research progress in musculoskeletal models, traditional machine learning models, and deep learning models applied in sEMG-based intention recognition. Among them, three representative deep learning models are selected, namely, convolutional neural networks (CNN), recurrent neural networks (RNN), and hybrid-structured models, as illustrated in Figure 2, showcasing some application examples.

#### 2.2.1. Musculoskeletal Models

Musculoskeletal models are the most commonly used model-based approaches for myoelectric prosthetic hand control. These models aim to understand how neural commands responsible for human motion are transformed into actual physical movements by modeling muscle activation, kinematics, contraction dynamics, and joint mechanics [63]. Incorporating detailed muscle–skeletal models in the study of human motion contributes to a deeper understanding of individual muscle and joint loads [64]. The earliest muscle model was first proposed by A. V. Hill in 1938. It was a phenomenological lumped-parameter model that provided an explanation of the input–output data obtained from controlled experiments [21]. Through the efforts of researchers, the muscle–skeletal models commonly used in the field of EMG prosthetic hand control include the Mykin [23] and simplified muscle–skeletal models [25].

Musculoskeletal models, which encode the explicit representation of the musculoskeletal system’s anatomical structure, can better simulate human physiological motion [65], and are, therefore, commonly applied in research on human motion intention recognition. However, for EMG prosthetic hand human–machine interfaces based on motion skeletal models, it is necessary to acquire sEMG signals corresponding to the muscles represented by the model. This may require the use of invasive electrodes, which inevitably pose physical harm to the subjects’ limbs and require the involvement of expert physicians, leading to various inconveniences and obstacles. Another promising approach to facilitate the wider application of muscle–skeletal models is the localization of specific muscles in the subjects’ limbs through high-density sEMG signal decomposition techniques.

#### 2.2.2. Traditional Machine Learning Models

Machine learning algorithms typically establish mappings between inputs and desired target outputs using approximated numerical functions [8]. They learn from given data to achieve classification or prediction tasks and are widely applied in the field of motion intention recognition.

Gaussian processes are non-parametric Bayesian models commonly applied in research on human motion intention recognition. Non-negative matrix factorization (NMF) is one of the most popular algorithms in motion intention recognition based on sEMG. As the name suggests, the design concept of this algorithm is to decompose a non-negative large matrix into two non-negative smaller matrices. In the field of motion intention recognition based on sEMG, the sEMG signal matrix is often decomposed into muscle activation signals and muscle weight matrices using non-negative matrix factorization algorithms. The muscle weight matrix is considered to reflect muscle synergies [29].

Despite achieving certain results, motion intention decoding models based on traditional machine learning algorithms often rely on tedious manual feature engineering. Research has shown that methods utilizing traditional machine learning algorithms still fail to meet the requirements of current human–machine interaction scenarios, such as EMG prosthetic hand control, in terms of accuracy and real-time responsiveness [66].

#### 2.2.3. Deep Learning Models

Deep learning algorithms can be used to classify input data into corresponding types or regress them into continuous sequences in an end-to-end manner, without the need for manual feature extraction and selection [9]. The concept of deep learning originated in 2006 [67], and, since then, numerous distinctive new algorithm structures have been developed. In this section, we discuss three commonly used deep learning methods for motion intention recognition: CNN and its variants, RNN and its variants, and hybrid-structured networks. We provide an overview of their research progress.

***CNN-based models***: CNN was first proposed in 1980 [68]. Due to its design of convolutional layers, it has the capability to learn general information from a large amount of data and provide multiple outputs. CNN has been applied in various fields such as image processing, video classification, and robot control. It has also found extensive applications in the field of motion intention recognition;

***RNN-based models***: The introduction of RNN was aimed at modeling the temporal information within sequences and effectively extracting relevant information between input sequences. However, due to the issue of vanishing or exploding gradients, RNN struggles to remember long-term dependencies [9]. To address this inherent limitation, a variant of RNN called Long short-term memory (LSTM) was introduced, which has gained significant attention in research on recognizing human motion intentions. Numerous studies have been conducted on the application of LSTM in this field;

***Hybrid-structured models***: Hybrid-structured deep learning algorithms typically consist of combining two or more different types of deep learning networks. For motion intention recognition based on sEMG, hybrid-structured deep learning algorithms often outperform other approaches in intention recognition tasks. One compelling reason for this is that hybrid-structured algorithms extract more abstract features from sEMG signals, potentially capturing more hidden information. This leads to improved performance in motion intention recognition.

Deep learning algorithms have been widely applied in the recognition of human motion intentions due to their unique end-to-end mapping approach. They eliminate the need for researchers to manually extract signal features and instead learn more abstract and effective features through the depth and breadth of their network structures. However, their lack of interpretability makes it challenging to integrate them with biological theories for convincing analysis. Moreover, the increased complexity of networks associated with improved recognition accuracy results in significant computational demands. This poses challenges for tasks such as EMG prosthetic hand control that require fast response times within specific time frames. Currently, most research in this area is based on offline tasks. Therefore, key technical research focuses on how to incorporate human biological theories into the design of deep learning algorithms and achieve high accuracy and fast response in motion intention recognition solely through lightweight network structures.

### 2.3. Mapping Parameters

Mapping parameters, as the output part of the motion recognition scheme, serve as the parameterization of user motion intention and control commands for the myoelectric prosthetic hand system. Human hand motion is controlled by approximately 29 bones and 38 muscles, offering 20–25 degrees of freedom (DoF) [69], enabling flexible and intricate movements. The movement of the human hand is achieved through the interaction and coordination of the neural, muscular, and skeletal systems [70]. This implies that the parameterization of motion intention should encompass not only the range of motion of each finger but also consider the variations in joint forces caused by different muscle contractions. In myoelectric hand control, two commonly used control methods exist [8]: (1) Using surface electromyography (sEMG) as the input for decoding algorithms, joint angles are outputted as commands for controlling low-level actuators; (2) Using sEMG as the input, joint torques are output and sent to either the low-level control loop or directly to the robot actuators (referred to as a force-/torque-based control algorithm). Based on the aforementioned analysis, this manuscript provides an overview of mapping parameters, which are categorized into three parts: kinematic parameters, dynamic parameters, and other parameters.

#### 2.3.1. Kinematic Parameters

Parameterizing human motion intention typically involves kinematic parameters such as joint angles, joint angular velocity, and joint angular acceleration:

***Joint angle***: Analyzing biomechanical muscle models reveals [45] that joint angles specifically define the direction of muscle fibers and most directly reflect the state of motion;

***Joint angular velocity***: If we consider joint angles as the state, then joint angular velocity serves as the control vector for changing the state, representing the rate of change of joint angles in the human body per unit time. Joint angular velocity is more stable compared to other internal physical quantities, making it advantageous for better generalization to new individuals. It is closely related to the extension/flexion movement commands of each joint [71];

***Joint angular acceleration***: Joint angular acceleration describes the speed at which the joint motion velocity in the human body changes. The relationship between joint angular acceleration and joint angular velocity is similar to the relationship between joint angles and joint angular velocity. Some studies suggest a significant correlation between joint angular acceleration and muscle activity [72].

#### 2.3.2. Dynamics Parameters

When using a myoelectric prosthetic hand to perform daily grasping tasks, the appropriate contact force is also a crucial factor in determining task success rate for individuals with limb loss. Among the dynamic parameters commonly used for parameterizing human motion intention, joint torque has been proven to be closely related to muscle strength [21]. For laboratory-controlled prostheses operated through a host computer, suitable operation forces can be achieved using force/position hybrid control or impedance control. However, for myoelectric prosthetic hands driven by user biological signals, effective force control of the prosthetic hand must be achieved by decoding the user’s intention. Therefore, the conversion of human motion intention into dynamic parameters is necessary.

#### 2.3.3. Other Parameters

A common approach to parameterizing human motion intention using specific indicators involves guiding the subjects to perform specific tasks to obtain target data and using these cues as labels [73]. Motion intention recognition is then achieved using supervised learning decoding models. Representing human motion intention using other forms of parameters to some extent reduces the complexity of intention decoding tasks, simplifying the process and facilitating the application of motion intention recognition schemes to practical physical platforms. However, when relying on end-to-end mapping established by decoding models, if the mapping variables lack meaningful biological interpretations, this further reduces the persuasiveness and interpretability of the already less interpretable decoding models. Therefore, when considering non-biological alternative parameter schemes, it is important to strike a balance between control performance and the interpretability of human physiological mechanisms.

## 3. Current Research Status

Research on myoelectric control for prosthetic hand manipulation is primarily focused on two aspects: the first is how to accurately decode human motion intention through a recognition algorithm, and the second is how to drive the prosthetic hand to perform that intention through a control strategy. Thus, the following sections provide a review of current research progress in intention recognition and control strategy for myoelectric prosthetic hands.

### 3.1. Advances in Intention Recognition Research

As a forward human–machine interaction component of the myoelectric prosthetic hand system, hand movement intention recognition based on sEMG aims to represent the user’s movement intention as specific physical parameters, which are then converted into control commands to achieve prosthetic hand movement control. Therefore, the following sections first summarize and classify the types of movement intentions involved in current research, and then review the discrete movement classification and continuous movement estimation methods separately.

#### 3.1.1. Type of Motion Intention

This section categorizes hand movement intentions based on task complexity by examining public datasets or self-built datasets of EMG signals. We have divided existing studies into two experimental paradigms: discrete motion performed separately and combined motion performed continuously.

***Discrete action performed separately***: The experimental paradigm of discrete action performed separately requires the subjects to repeat a certain action multiple times in a single experiment, and the hand needs to return to the initial state between actions, making it easier to mark data segments while facilitating the learning of intention recognition models. This is currently the most explored type of movement intention, including widely used publicly available datasets such as Ninapro [74], which were designed based on this paradigm. In addition to basic unconstrained discrete hand movement recognition, some studies have attempted to explore constrained discrete action recognition involving object manipulation [12], aiming to achieve more natural and intuitive control of myoelectric prosthetic hands. Discrete action performed separately treats the initial state as a transition stage between action data, similar to normalizing the data, making the data distinction between different actions higher. However, it ignores the switching between actions [9], leading to high offline recognition accuracy but errors in online control during the transition stage, resulting in less-than-ideal results when applied to myoelectric prosthetic hand control in practical applications.

***Combined action performed continuously***: In the experimental paradigm of combined action performed continuously, there is no strict requirement for subjects to return to the initial state between two actions, allowing for more natural execution of different hand movements, combinations, or transitions. Some researchers have even attempted to explore allowing subjects to perform actions that were not previously defined based on their own will [27]. The continuous execution of combined actions undoubtedly obtains more human motion data that are closer to real-life activities. However, the difficulty of intention recognition tasks will also increase significantly. This is not only because there are more diverse types of actions, but also because the transitions between similar actions or the combinations of different actions make the recognition task more complex. Therefore, current researchers are still exploring how to effectively recognize the movement intentions of different action combinations or transitions [37].

#### 3.1.2. Discrete Motion Classification

Discrete action treats the entire process of the user performing a hand movement as a single pattern, simplifying the representation of hand movement through static action by ignoring changes in hand structure during motion. Currently, the most advanced method for achieving discrete action classification is pattern recognition technology. Based on pattern recognition, discrete action classification tasks are designed according to predefined action categories, and corresponding motion labels are created for the sEMG data of each action. For new sEMG signals, the motion label with the highest similarity based on its data characteristics is assigned to complete the action classification task.

Several studies have reviewed the research progress on implementing discrete action classification tasks based on pattern recognition [75,76]. For discrete action classification tasks, the number of predefined motion types is crucial for achieving intuitive myoelectric prosthetic hand control. Hence, we select studies with more than 40 predefined motion types as the latest research advancements in this field. Parviz Ghaderi et al. [77] proposed three new sEMG features based on kernel density estimation to improve the classification accuracy of a large number of hand movements. They achieved an accuracy of 98.99 ± 1.36% in classification of 40 hand and wrist movements involving 40 able-bodied and 11 amputee subjects. Pizzolato et al. [78] conducted a comparative experiment on 6 existing data acquisition devices for 41 hand movement classification tasks. Although the best accuracy rate achieved was only 74.01 ± 7.59%, this study provides a reference solution for small-scale laboratories and pediatric prosthesis, given the cost constraints. Panyawut et al. [79] achieved high-precision recognition of 41 hand and wrist movements based on sEMG signals using deep neural networks, with an accuracy rate of up to 90%. According to their results, this is currently the study with the largest number of predefined motion types with an accuracy rate greater than 90%. Zhai et al. [80] implemented an effective self-recalibration function for myoelectric control by combining a CNN classifier with a simple label updating mechanism. Despite achieving a highest accuracy of only 61.7% in a 50-class hand gesture classification task, the label updating mechanism improved the classifier’s accuracy by 4.2 to 10.18%.

Implementing discrete action classification tasks based on pattern recognition reduces the difficulty of the task by simplifying the representation of hand movements while significantly improving the accuracy of traditional motion intention recognition schemes. However, this method itself has inherent drawbacks. Discrete action classification tasks rely on predefined labels assigned to the data and do not have actual physical meaning. In fact, the supervised learning architecture that depends on predefined labels lacks the ability to generalize to undefined categories [81]. In addition, experiments in laboratory environments usually only consider the static part of sEMG signals (time intervals where force is maintained at roughly constant levels without movement) for classification. However, the transitions between different movements are characterized by non-stationary signal components [3]. This is also the main reason for errors occurring in practical applications of pattern recognition-based classification systems. Therefore, predicting the transitional states between gestures based on pattern recognition methods is a key issue that needs to be urgently addressed.

#### 3.1.3. Continuous Motion Estimation

In contrast to discrete action classification, continuous motion estimation tasks aim to capture the entire process of user hand movements, including the different completion stages of a single action and the transitional stages between different actions. This is a more complex dynamic process. Continuous motion estimation tasks do not rely on predefined action patterns, but instead estimate the hand state in real-time at a single moment, such as joint angles, positions, or torques. This feature allows for more intuitive and natural control in myoelectric prosthetic hands, making continuous motion estimation tasks a research hotspot in the field of myoelectric intention recognition in recent years.

Most research in continuous motion intention recognition begins by validating the feasibility of single DoF motion estimation tasks, before gradually improving to tasks that estimate two or more DoF. Kapelner et al. [31] used the discharge time of MUs determined by decomposing high-density sEMG to predict three-DoF joint angles of the wrist, separately. Their results among seven participants demonstrated that the neural features obtained from sEMG decomposition outperformed traditional time-domain features in motion estimation. Dai et al. [82] achieved motion estimation for the MCP joint of a single finger during movement in flexion and extension manners continuously without interruption. They improved the performance of the regression method for simulating natural finger movements by combining array sEMG sensors and independent component analysis. He et al. [60] extracted muscle synergies from sEMG data to achieve single-DoF joint angle prediction for the wrist, thumb, index finger, and middle finger. Their results demonstrated higher prediction accuracy compared to traditional musculoskeletal models and machine learning methods.

The complex and flexible human hand system largely benefits from its flexible five-finger multi-joint structure. Therefore, the simultaneous estimation of multiple-DoF motion is crucial for decoding hand motion intentions and is an inevitable trend in the development of motion intention recognition research. Yu Yang et al. achieved simultaneous estimation of two-DoF wrist motion by constructing 2D images of sEMG signals that involved the globally spatial information across channels [46], and their subsequent research further enhanced wrist torque estimation accuracy by introducing interactions between different motion units to construct specific motion unit images [35]. Zhang et al. [12] proposed a sparse pseudo-input Gaussian process regression method to achieve simultaneous estimation of the five-DoF motion of the MCP joint in functional grasping tasks. This method is beneficial for intuitive and accurate myocontrol of robotic hands. Yang et al. [33] successfully decoded complex wrist movements with three-DoF directly from raw sEMG signals. Their findings demonstrated the high accuracy of this method (superior to support vector regression) and its generalizability (able to be extended to able-bodied subjects without specific training).

The successful application of motion intention recognition methods in multi-DoF simultaneous motion estimation tasks represents a significant advancement in the study of the complex structure and flexible functionality of the human hand. This provides a theoretical foundation and feasibility for restoring hand function in amputees through prosthetic hands. However, due to the non-stationarity of sEMG signals and the complexity of and variability in human motion, the intrinsic physiological–physical relationship between sEMG and hand movements cannot be fully described [83], which leads to high difficulty in implementing continuous motion estimation. The estimated physical quantities are only approximations, and accuracy still needs to be improved.

### 3.2. Advances in Control Strategy Research

**Figure 3:**
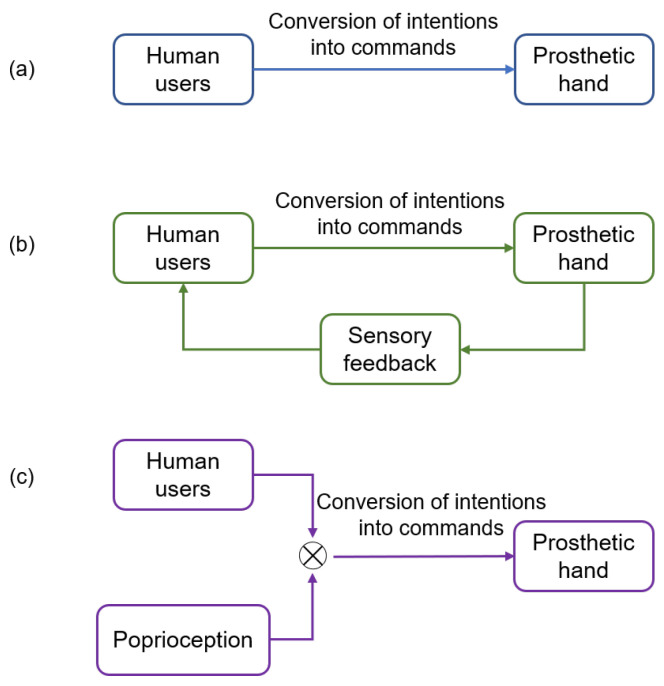
Control strategy categories. (**a**) Unidirectional control strategy. (**b**) Feedback control strategy. (**c**) Shared control strategy.

The intention decoder only focuses on accurately recognizing the user’s motion intentions. The research on control strategies is mainly focused on how to efficiently and intuitively execute the recognized intentions using myoelectric prosthetic hands. Based on the complexity of the control framework, current research on control strategies can be divided into three categories: unidirectional control, feedback control, and shared control, as shown in Figure 3. In this manuscript, we provide an overview of the progress made in each of these areas.

#### 3.2.1. Unidirectional Control

Unidirectional control involves single-directional control of the myoelectric prosthetic hand, with the user’s motion intention as the sole control source. This can be achieved by directly mapping signal amplitude or signal characteristics to the activation or degree of motion of the prosthetic hand. This control strategy is the most straightforward and most widely used, and its effectiveness has been validated in commercial products [84,85,86].

Hahne et al. [32] have successfully applied regression methods to the unidirectional control of myoelectric prosthetic hand and explored the impact of different arm positions and time restrictions. The proposed method outperformed two clinical control methods in most cases and demonstrated robust performance over multiple days on five prosthetic users. Similarly, Domenico et al. [87] investigated the superiority of nonlinear regression classifiers for myoelectric unidirectional control, conducting experiments in which amputee subjects intuitively and simultaneously controlled the Hannes system. Pizza et al. [20] successfully combined probability-weighted regression with sEMG signals to simultaneously control the multiple DoF of prosthetic hands. The algorithm demonstrated excellent performance in the two-DoF case and enabled amputees to perform several daily tasks using a two-DoF wrist prosthesis. Lukyanenko et al. [14] proposed a stable unidirectional control strategy for myoelectric prosthetic hands based on a collaborative framework, achieving long-term use in 3-DOF control for up to 10 months and in 4-DOF control for up to 9 months.

In summary, the research on unidirectional control strategies for myoelectric prosthetic hands has been focused on the practical application of regression techniques to clinical practice with the aim of increasing the number of DoF that users can control simultaneously. The performance of the unidirectional control strategy is constrained by the insufficient sensory information available to the user, with users primarily relying on visual guidance and utilizing residual limb proprioception as a weak auxiliary aid. However, overreliance on vision can limit the control of prosthetic hands, which is one of the reasons for high abandonment rates of commercial prosthetic hands.

#### 3.2.2. Feedback Control

Sensory feedback in prosthetic hands is a hot topic in current research that has experienced a sharp increase in the number of studies conducted in the past few years. Although previous studies have claimed that feedback control does not significantly improve performance or only enhances the performance of prosthetic hands in specific contexts [88], recent years have seen substantial research progress as researchers delve deeper into the fundamental role of feedback in prosthetic hand control. Due to inherent risks, there have been relatively few studies focused on invasive sensory feedback. This section primarily focuses on a review of non-invasive sensory feedback systems, such as mechanotactile, vibrotactile, electrotactile, and their combinational systems.

Several prior reviews have extensively summarized the methods employed in providing feedback to users of prosthetic hands [88,89,90]. Hence, we focus on the latest progress in the successful application of feedback control to prosthetic hand systems. Xu et al. [91] demonstrated the restoration of finger-specific tactile sensation through electrical stimulation, enabling amputees to successfully discriminate between finger-pressing states, object curvatures, and hardness. Their results demonstrated that amputee subjects were able to discriminate objects with varying curvature and hardness with an accuracy of over 90%. Shehata et al. [92] explored the use of an audio-guided feedback control strategy, which was found to be superior to the unidirectional control strategy in terms of path efficiency and other parameters. Their results from experiments demonstrated that the use of enhanced feedback control can improve both short-term and long-term performance of prosthetic hands. Li et al. [93] developed a portable electrical tactile stimulator and applied it in a virtual grasp scenario using myoelectric control. Their experimental results indicated that the success rate of grasping with electrical tactile feedback control was higher than that of the unidirectional control strategy. During heavy object grasping experiments, users are required to exert less effort, thereby effectively alleviating muscle fatigue associated with task performance. Cha et al. [94] proposed a new method for providing feedback on grasp information for robotic prosthetic hands and built a closed-loop integrated system for experimental validation consisting of an EMG classification model, the proposed tactile device, and the robotic prosthetic hand. The experimental results demonstrated that the application of this new feedback method could improve the recognition accuracy of proprioceptive feedback and had the potential to be applied in the feedback control of prosthetic hands.

The aforementioned studies have achieved closed-loop control of prosthetic hands through the integration of myoelectric control interfaces and artificial feedback. Their results show that feedback control not only improves the performance and practicality of prosthetic hands, but also enhances the user’s experience during interaction with their bionic limb. However, the benefits of feedback control have mainly been demonstrated in laboratory conditions rather than in daily operating scenarios, thus lacking sufficient reliability. In addition, integrating non-invasive feedback devices remains a challenge to be overcome, as their large size and the need to wear and remove them daily may be detrimental to the user experience.

#### 3.2.3. Shared Control

For precise and repetitive tasks in structured environments, automated systems have higher processing efficiency. In unstructured environments, however, humans possess the ability to make quick judgments and adapt flexibly. However, due to the limited capacity and energy of humans, automated systems and humans need to collaborate in task execution. This mode of collaboration is known in the fields of robotics and neural engineering as “shared control”. The concept of shared control has been applied in many research fields, such as teleoperated robots [95,96], brain–machine interfaces [97,98], autonomous driving [99], and surgical assistive robots [100]. According to the latest review on shared control schemes applied to teleoperated robots [101], existing shared control schemes can be classified into semi-autonomous control (SAC), state-guided shared control (SGSC), and state-fusion shared control (SFSC) based on the different ways of sharing between human users and autonomous controllers. Although SGSC and SFSC, which offer richer interaction means and more intelligent operations, are the future development trend, SAC, which is relatively simple but more practical, more user-friendly, and better aligned with the design intention of shared control schemes to combine the advantages of human users and intelligent controllers, remains more popular and preferred for enhancing task performance and reducing user burden.

The application scenarios of prosthetic hands include both repetitive and intricate daily activities, as well as dexterous manipulation tasks. Therefore, prosthetic hands need to adopt a shared control approach to work collaboratively with human users. In this mode, prosthetic hands can respond to user commands and movements to achieve more natural control and higher precision. SAC is also the most commonly used shared control scheme in the field of prosthetic hands, and researchers have proposed a series of shared control schemes based on different perceptual methods. In this section, we mainly investigate the current state of research in visual perception and tactile perception.

Visual perception of prosthetic hands can provide information about the shape of objects, allowing autonomous controllers to select grasping types or adjust finger configurations accordingly. Shared control schemes for visual perception include the following. Mouchoux et al. [102] proposed a novel AR feedback semi-autonomous control scheme that not only improved the flexibility of EMG control, but also effectively improved user experience by shortening operation time and reducing muscle activity. Castro et al. [103] designed a shared control scheme by placing a depth sensor on the back of the prosthetic hand, which enabled online interaction between users and the prosthetic hand. Users were responsible for aiming at the whole or part of the object, and the control system continuously responded to the aimed target. Starke et al. [104] proposed a semi-autonomous control scheme based on visual object recognition to automatically select and execute grasping trajectories and wrist orientations. Their results indicated that, compared to traditional myoelectric control, the vision-based shared control strategy enabled a faster and less physically demanding grasping process. Federico et al. [105] developed an approach based on “hand-eye learning” to control hand pre-shaping and grasp the aperture before grasping, according to the input from a wrist-mounted camera. They considered different types of grasping that can be associated with different parts of objects and successfully achieved complete control effects on the Hannes prosthetic hand.

Tactile perception of prosthetic hands can assist in achieving functions such as contact detection, adaptive object shape, and force closure. Relevant research includes the following. Cipriani et al. [106] compared three types of tactile perception shared control schemes: fully autonomous control, semi-autonomous control, and direct user control, and found that users preferred the semi-autonomous control scheme, even though the control performances were about the same. Zhuang et al. [107] designed a shared control scheme based on tactile sensors placed on the inside of the prosthetic hand. The prosthetic hand performed the grasping action based on user intention and then automatically maximized the contact area between the hand and the object according to tactile feedback, effectively enhancing user endurance while assisting in achieving stable grasping. Seppich et al. [108] placed tactile sensors at the end of the prosthetic to perceive the shape and hardness of the object and provide feedback to the user to improve task performance. Their results demonstrated that the proposed approach successfully assisted amputees in performing tasks of screwing in a lightbulb and flipping cups. Mouchoux et al. [109] used a pressure sensor placed on the thumb to detect contact with the target object, enabling the autonomous controller to judge the current task execution stage and implement shared control of the prosthetic hand based on three different user intentions and the decision of the autonomous controller.

Shared control schemes aim to bridge the gap between user intention and task execution expectations by combining the user’s motion intention and decisions made by the prosthetic hand’s own perception. This can effectively overcome the inherent limitations of muscle myoelectric control schemes that rely solely on user intention. Moreover, these schemes significantly relieve the user’s burden, compensate for insufficient neural muscle function, and enhance the robustness of myoelectric control. The use of shared control has significant implications for the field of prosthetic hands.

## 4. Challenges and Opportunities

The above summary of the current state of myoelectric control research highlights a range of state-of-the-art technologies and applications. Although significant experimental results have been achieved, there are still the following challenges that need to be addressed:

- Although there has been progress in decoding motion intentions for a variety of basic hand movements, there is a lack of functional motion intention decoding that facilitates prosthetic hand manipulation, which means that current prosthetic hands are only able to perform simple grasping tasks;
- Existing myoelectric control research primarily focuses on basic hand grasping functions in humans (see Table 2), whereas more investigations are required to explore complex daily manipulation tasks that demand continuous manipulation and dynamic grasping force adjustment;
- Prioritizing recognition and generalization capabilities while neglecting the high abandonment rate and subjective user experience of prosthetic hands is a flawed approach. During the processes of both myoelectric training and control, users need to exert a significant amount of attention and effort.

Therefore, we propose two potential research directions to address the aforementioned challenges, namely, functionality-augmented prosthetic hand and user burden reduction.

### 4.1. Functionality-Augmented Prosthetic Hands

Functionality-augmented prosthetic hands aim to restore basic hand functionality for amputees while also introducing new convenient features. Due to the complex mechanical structures and control methods, even the most advanced technology currently available cannot design prosthetic hands that are identical to human hands. As an advanced intelligent robot technology closely integrated with the human body, we believe that prosthetic hands should not only focus on restoring basic human hand functionality, but also on adding intelligent functions that only robotic hands can provide, rather than simply pursuing a perfect match to human hand functionality. This can not only restore the daily manipulation abilities lost by amputees, but also enable them to complete specific tasks more efficiently or even accomplish tasks beyond human hand functionality. Previous studies have indicated that enhanced hand functionality can have an impact on user hand neural representation and motor control abilities, with the potential for increased flexibility in use, reduced cognitive reliance, and increased proprioceptive sensation [115].

Frey et al. [116] presented an octopus-inspired bio-inspired neural system that can detect objects and automatically initiate adhesive contact. The proposed method was applied to a wearable glove for picking up and releasing various shaped underwater objects, including flat, curved, rigid, and soft objects. Chang et al. [117] introduced a humanoid prosthetic hand inspired by efficient swinging dynamics, which allowed users to control and maximize swinging velocity using the bio-inspired wrist mechanism design. Their results indicated that the proposed approach could increase the speed of the swing action by 19% at 90 rpm, meeting the demands of high load and high-speed swinging sports activities. However, for research on augmenting robot hand functionality, achieving coordinated motion control of the palm and fingers seems to be more attractive to users. Lee et al. [118] have proposed a novel robot palm with a dual-layered jamming mechanism, which automatically solidifies the palm by sensing internal pressure in the palm, enhancing grasping ability. Their results showed that, compared to a single-layer structure, the proposed palm could increase the contact surface area by 180% and increase the gripping force by 2–3.1 times. Heo et al. [119] proposed a bio-inspired triple-layered skin robot palm based on a porous latex structure; their results showed that the bio-inspired skin palm could firmly grasp the object while expanding the contact area due to its unique stiffness properties, demonstrating stronger grasping function and robustness to external interference in grasping tasks.

In summary, functionality-augmented prosthetic hands are one of the effective methods to overcome the current limitations of single-functionality in myoelectric prosthetic hands and are expected to bring more efficient and intelligent control for amputees. However, there are several challenges that functionality-augmented prosthetic hands still need to overcome before they can be widely applied:

- The activation method of functionality-augmented technology must be intuitive and natural. If it requires complex pre-actions from the user, it will significantly increase their cognitive and control burden, such as requiring extensive long-term training. The multimodal human–machine interface for prosthetic hands may be an effective solution to this challenge [120,121]. It uses sEMG as the primary signal source, with other biological signals from the hand used as an auxiliary signal source to achieve natural and implicit control of functionality-augmented technology;
- The hardware equipment that provides functionality-augmented technology needs to be highly integrated, ensuring that the overall volume and weight of the prosthetic hand remain within an acceptable range for the user;
- Hand function augmentation may cause changes in the biological hand representation of the user, which is also a problem that needs to be addressed in functionality-augmented prosthetic hands. Functionality-augmented technology should not affect the user’s ability to control basic hand functions. Instead, it should produce a beneficial gain in the user’s own motion control capability, rather than a confusing adverse effect.

### 4.2. User Burden Reduction

The two key factors influencing the acceptance rate of prosthetic hands are intuitive control experiences and user burden [122,123]. However, current researchers focus more on how to develop myoelectric classifiers that include diverse gestures, rather than paying enough attention to the training and control burdens of users. We believe that reducing the training and control burdens on users is beneficial for improving the high abandonment rate of prosthetic hands in society and promoting the clinical application of myoelectric prosthetic hands by enhancing user subjective experience. The user control burden can be effectively reduced through intelligent control strategies, as detailed in Section 3.2. Therefore, the following discussion focuses on the user training burden.

Generally, myoelectric training data acquired under ideal laboratory conditions yield promising results in offline experiments. However, they often perform poorly in actual prosthesis applications due to the influence of hybrid factors in real-world scenarios [9]. These hybrid factors include non-ideal condition factors such as limb posture changes, skin perspiration, terminal load, muscle fatigue, long-term variations, or electrode shifts. Researchers have developed corresponding remedial measures based on different non-ideal condition factors: updating myoelectric models based on transfer learning to overcome electrode displacement or individual differences [124,125], enriching training datasets to address posture changes [126], and extracting EMG decomposition and synergistic features to overcome muscle fatigue [127,128], among others. A comprehensive review by Ziyou Li et al. [4] provides insights into the progress of myoelectric research under non-ideal conditions. This results in unsatisfactory myoelectric control despite heavy data acquisition tasks carried out by participants. Therefore, improving the quality and efficiency of training data acquisition tasks is crucial for reducing the training burden on users.

Improving myoelectric training paradigms is an effective method for reducing the user training burden. Dapeng Yang et al. [129] proposed a dynamic myoelectric training paradigm that explored the effects of upper limb movement, contraction level, and unintentional EMG activation on training data. Their results showed that the training paradigm, which involved dynamic upper limb postures and dynamic muscle contractions, achieved the most accurate and robust classifier. Morten et al. [130] explored the effects of restricting wrist and hand movements in able-bodied subjects to bridge the performance gap in myoelectric control between able-bodied and amputee subjects, and also tested the influence of arm posture. The experimental results suggested that restricting healthy limb movements is an effective training paradigm for improving training efficiency and reducing performance differences between able-bodied and amputee subjects. In addition, there have been similar paradigm improvement studies for other biosignal-driven prosthetic hands that have reference value for myoelectric training paradigms. Susannah et al. [131] explored the effects of different socket loads, arm positions, and motion patterns on training paradigms and verified, for the first time, the feasibility of using sonomyography to control prosthetic hands. Jiarong Wang et al. [132] improved the classic center-out paradigm in the field of EEG signal research, enhancing the training paradigm’s movement prediction performance and generalizability, significantly reducing subjects’ physical exertion.

In conclusion, we believe that improving myoelectric training paradigms is a research direction that needs to be vigorously pursued in the future. In comparison to myoelectric classifiers that require tens of hours or even days to obtain a rich set of gestures, users prefer classifiers that have a smaller training burden and are more stable. Additionally, we believe that exploring training paradigms based on continuous hand movements, rather than the traditional paradigm of extracting discrete movements, is a feasible direction for improving the manipulation performance of myoelectric control.

## 5. Conclusions

In this study, we conducted a comprehensive investigation of recent advances in myoelectric control for prosthetic hand manipulation, with a primary focus on intention recognition and control strategy research. We first briefly introduced the basic concepts of myoelectric control, including sEMG signal processing, decoding models, and mapping parameters, which are three important procedures. Then, we reviewed the current advances in intention recognition research from three perspectives: motion intent types, discrete motion classification, and continuous motion estimation. In addition, we also reviewed the current advances in control strategy research from three aspects: unidirectional control, feedback control, and shared control. Based on the above review, we proposed two future research directions that can overcome the current limitations of myoelectric control, namely, functionality-augmented prosthetic hands and user burden reduction. Improving the myoelectric control performance for prosthetic hand manipulation is beneficial for enhancing the clinical application potential of myoelectric prosthetic hands and increasing their widespread usage.

## Figures and Tables

**Table 1:** Typical application examples of each module.

Module	Types	Reference
Feature engineering	Single time-domain feature	MAV [12,13,14], RMS [15,16,17]
	Combined time-domain features	RMS+WL+ZC [18], ZOM+SOM+FOM+PS+SE+USTD [19], MAV+WL+ZC+SSC+SOAMC [20]
Decoding model	Musculoskeletal model	Hill-type muscle model [7,21,22], Mykin model [23,24], Lumped-parameter model [25,26]
	Traditional machine learning model	Gaussian processes [12,27,28], NMF [15,29,30], Linear regression [31,32]
	Deep learning model	CNN-based model [33,34,35,36], RNN-based model [37,38,39], Hybrid-structured model [40,41,42]
Mapping parameters	Kinematic parameters	Joint angle [12,17,43], Joint angular velocity [28,44], Joint angular acceleration [39,45]
	Dynamics parameters	Joint torque [35,46,47,48]
	Other parameters	3D coordinate value [49,50], Movement of the in-hand object [51], Multidimensional arrays [14], Movement activation level [52]

MAV: Mean absolute value, RMS: Root mean square, WL: Waveform length, ZC: Zero crossing, SSC: Slope-sign changes, SOAMC: Sixth order autoregressive model coefficients, ZOM: Zero order moment, SOM: Second order moment, FOM: Fourth order moment, PS: Peak stress, SE: Shake expectation, USTD: Unbiased standard deviation, NMF: Non-negative matrix factorization.

**Table 2:** Summary of current application scenarios for prosthetic hand manipulation.

Manipulation Scenarios	Tasks	Reference
	Grasp test	[81,110,111,112]
	Box-and-blocks test	[32,107,113]
Simple manipulation tasks	Relocation test	[32,102,103]
	Pouring or drinking	[20,49,107]
	Screwing test	[49,108]
	Block building	[49]
Complex manipulation tasks	Squeezing toothpaste	[20]
	Bimanual interaction	[114]

## Data Availability

Data sharing not applicable.
